# Association Study of 25 Type 2 Diabetes Related Loci with Measures of Obesity in Indian Sib Pairs

**DOI:** 10.1371/journal.pone.0053944

**Published:** 2013-01-17

**Authors:** Vipin Gupta, Donipadi Guru Vinay, Ulla Sovio, Sajjad Rafiq, Madamchetty Venkata Kranthi Kumar, Charles Spurgeon Janipalli, David Evans, Kulathu Radha Mani, Madana Narasimha Sandeep, Amy Taylor, Sanjay Kinra, Ruth Sullivan, Liza Bowen, Nicholas Timpson, George Davey Smith, Frank Dudbridge, Dorairaj Prabhakaran, Yoav Ben-Shlomo, Kolli Srinath Reddy, Shah Ebrahim, Giriraj Ratan Chandak, the Indian Migration Study Group

**Affiliations:** 1 South Asia Network for Chronic Disease, Public Health Foundation of India, New Delhi, India; 2 Centre for Cellular and Molecular Biology (CCMB), Council of Scientific and Industrial Research (CSIR), Hyderabad, India; 3 Faculty of Epidemiology and Population Health, London School of Hygiene and Tropical Medicine, London, United Kingdom; 4 University of Southampton, Southampton, United Kingdom; 5 School of Social and Community Medicine, University of Bristol, Bristol, United Kingdom; 6 MRC Centre for Causal Analyses in Translational Epidemiology, University of Bristol, Bristol, United Kingdom; 7 Centre for Chronic Disease Control, New Delhi, India; 8 Public Health Foundation of India, New Delhi, India; Sanjay Gandhi Medical Institute, India

## Abstract

Obesity is an established risk factor for type 2 diabetes (T2D) and they are metabolically related through the mechanism of insulin resistance. In order to explore how common genetic variants associated with T2D correlate with body mass index (BMI), we examined the influence of 25 T2D associated loci on obesity risk. We used 5056 individuals (2528 sib-pairs) recruited in Indian Migration Study and conducted within sib-pair analysis for six obesity phenotypes. We found associations of variants in *CXCR4* (rs932206) and *HHEX* (rs5015480) with higher body mass index (BMI) (β = 0.13, p = 0.001) and (β = 0.09, p = 0.002), respectively and weight (β = 0.13, p = 0.001) and (β = 0.09, p = 0.001), respectively. *CXCR4* variant was also strongly associated with body fat (β = 0.10, p = 0.0004). In addition, we demonstrated associations of *CXCR4* and *HHEX* with overweight/obesity (OR = 1.6, p = 0.003) and (OR = 1.4, p = 0.002), respectively, in 1333 sib-pairs (2666 individuals). We observed marginal evidence of associations between variants at six loci (*TCF7L2, NGN3, FOXA2, LOC646279, FLJ3970* and *THADA*) and waist hip ratio (WHR), BMI and/or overweight which needs to be validated in larger set of samples. All the above findings were independent of daily energy consumption and physical activity level. The risk score estimates based on eight significant loci (including nominal associations) showed associations with WHR and body fat which were independent of BMI. In summary, we establish the role of T2D associated loci in influencing the measures of obesity in Indian population, suggesting common underlying pathophysiology across populations.

## Introduction

With more than 300 million sufferers worldwide [1] and strong relation with multiple chronic diseases, including type 2 diabetes (T2D), hypertension and coronary heart disease [2], obesity is a major public health problem. Association of obesity with T2D is widely demonstrated in western population [3] and is also well established for Asians [4]. The prevalence of obesity and T2D are rising simultaneously in Asia [5], which might be a coincidence or is likely the result of obesity leading to insulin resistance and T2D risk [6] modulated by body mass index (BMI) [7]. It is also possible that they have common biology, which could be seen as T2D possibly driving obesity, for instance, diabetes related loci are known to influence birth weight [8] and development of fat tissue [9], [10]. In India, genetic association studies related to obesity are few [11], [12], [13] and even fewer exist for related quantitative phenotypes [14], [15]. Several novel loci associated with obesity and its related quantitative phenotypes [e.g. BMI, waist hip ratio (WHR) and weight] have been identified through genome-wide association studies (GWAS) [16], [17]. Further, these novel single nucleotide polymorphisms (SNPs) related to obesity are also known to increase the risk of T2D in Europeans [18]. Similarly, loci identified from GWAS of T2D [19] and glycemic traits [20] have also been investigated for their associations with obesity and BMI. Recently, several loci influencing fasting insulin levels were reported to be associated with BMI and with WHR [21] in Europeans, which is consistent with the observation that higher BMI is associated with insulin resistance [22]. Such studies that have explored T2D loci and obesity related traits are few in Indian population. Moreover, to date, no GWAS of obesity-related traits and T2D has been conducted on individuals of Indian origin [23], [24].

We hypothesized that there could be variance in BMI that could be explained due to shared biological underpinning attributable to T2D. We had previously investigated 25 T2D related loci in subjects from “Indian Migration Study” (IMS) and found association of seven SNPs with various glycemic traits [25]. The same SNPs were also investigated in a study from India, which reported associations of sixteen SNPs with T2D and related intermediate traits [15]. In the present study, we assessed the role of these 25 SNPs that were previously identified by GWAS related to T2D in Europeans, with six obesity related phenotypes [i.e. BMI, overweight (BMI≥25), WHR, percentage body fat (body fat), weight and waist circumference (WC)].

## Methods

### Study Population

The present study included data on participants recruited in the population based “Indian Migration Study” conducted (during 2005–2007) in factories located in four cities of India (Lucknow, Nagpur, Hyderabad and Bangalore) [26]. Briefly, each migrant factory worker and his/her spouse were asked to invite one non-migrant full sibling of the same sex and closest to them in age still residing in their rural place of origin. A total of 7067 individuals were recruited in the Indian Migrant Study (IMS), of which 6780 were true siblings (3390 pairs), 272 unrelated pairs (cousins and friends; 136 pairs) and 15 individuals were singletons. Out of 3390 true sibling pairs, final analysis for quantitative traits was done on 2528 sib-pairs (5056 individuals) after excluding pairs: a) with missing genotyping data on >50% (n = 301 pairs) of the 25 genotyped SNPs in either of the siblings and b) with single/both T2D (n = 561 pairs) to avoid phenotypic heterogeneity which could cause distorted relationship with anthropometric traits [19]. We were left with 1333 sib-pairs (2666 individuals) concordant/discordant for overweight after excluding pairs with one or both sibs having T2D. Those with BMI ≥25 kg/m^2^ were classified as overweight [27]. Ethical approval was obtained from the ethics committee of the All India Institute of Medical Sciences, New Delhi, India (AIIMS; reference number A-60/4/8/2004). Written consent was obtained from all the study participants of IMS.

### Anthropometry and Diet

Weight was measured in light indoor clothing (with shoes removed) using a digital weighing scale with an accuracy of 100 g (Model-PS16, Beurer, Germany). Standing height was measured using a portable plastic stadiometer (Leicester height measure, Chasmors Ltd, London) [26]. Waist and hip girths were measured using a non-stretch metallic tape with a blank lead-in (Chasmors metallic tape, Chasmors Ltd, London). We calculated the percentage body fat, a measure of body composition, using standard formulae from triceps and subscapular skinfold measures using Holtain calipers [28]. Weight, height and circumferences were measured twice and skinfolds were measured thrice and mean of the repeated measures was taken for all the traits. The acceptable difference between two readings of variables was less than equal to 0.5 cm for height, 0.5 kg for weight, 0.5 cm for circumferences and 0.1 mm for skin folds. Average daily intake of energy consumption (kcal/day) was estimated using food frequency questionnaire (FFQ), which inquired participants about consumption (daily, weekly, monthly or yearly) of 184 different food items. Metabolic equivalent tasks (MET) scores were calculated from participants’ accounts of their activities in the previous month. Data of their daily activities were summarized as MET hours per day where 1 MET was equal to energy expended whilst sitting quietly. The average daily MET scores based on time spent on moderate to vigorous activities were calculated for each individual (defined as: moderate 3–6 MET; vigorous *>*6 MET) in a 24-hour period.

### Genotyping and Quality Control

In order to get independent and/or overlapping effects of T2D SNPs on obesity related traits, we evaluated data from all the 25 T2D SNPs generated (in 2009–10) using Sequenom Mass Array as part of a common multiplex pool. The study was started in 2008 and included GWAS SNPs published at that time [29], [30], and also included T2D associated SNPs having p-values>10^−7^ (see Table S2). The genotyping success rate was >95% and duplicate samples had >97% concordance.

### Sample Size and Power Calculation (see Table S3)

The power estimates were derived using the Genetic Power Calculator using option “QTL association for sibships and singletons” [31]. Given a sample size of 2528 sib pairs (5056 individuals) and minor allele frequency (MAF) ranging from 10% to 45%, our study had 80% power, at α = 0.05, to detect a quantitative trait locus explaining 0.7% of variation in each of the tested traits. For overweight, sample size of 1333 pairs (2666 individuals), MAF of 10% and 45% provides the power of >80% for detecting OR = 1.5 and OR = 1.3, respectively, at α = 0.05 [QUANTO-1.1 (http://hydra.usc.edu/gxe) option “case-sibling”]. Two different software packages were used because the desired options for sib pair design for quantitative and qualitative traits together were not available in any one of them.

### Statistical Analyses

Hardy Weinberg equilibrium (HWE) was estimated on unrelated individuals residing in an urban location i.e. a single randomly chosen member from each pair (N = 2528 out of 5056 individuals i.e. 2528 sib pairs, see Table S1) using exact test implemented in PLINK (version 0.99p; http://pngu.mgh.harvard.edu/~purcell/plink) [32]. All continuous variables were normally distributed and were transformed into z scores prior to analysis for the interpretation of regression coefficient in terms of change in standard deviation (SD) per allele. For all the traits, association analysis was carried out using the orthogonal family-based model [33] assuming additive model of inheritance using STATA (version 10). This is a mixed regression model in which the genetic effect is decomposed into fixed between- and within-family effects, with inference made on within-family effect. We applied multi-level model(s) adjusted for age, sex and location (i.e. city) for analyses on all quantitative traits because these covariates were associated with obesity in the study population [26]. Similar regression models were applied for analysis on overweight.

Since, some of the common genetic variants related to obesity are thought to have effect through the mechanism of appetite to maintain greater adiposity [34], we also adjusted all the traits including overweight for daily energy consumption (kcl/day) and physical activity (i.e. average daily MET score) but this did not affect the results (Table S4 and S5). Inferences were made on the basis of corrected alpha values using false discovery rate (FDR) [35] for overweight (0.004), BMI (0.004), WC (0.002), WHR (0.002), body fat (0.002) and weight (0.004). Despite strong evidence of the biological relationship between T2D and obesity, we used stringent p-values corrected for 25 SNPs for making inferences because there was no/weak prior evidence of their association with obesity related traits. For exploring the combined effect, the risk scores were estimated using eight loci [*CXCR4, HHEX, FOXA2, NGN3, TCF7L2, FLJ39307, LOC646279* and *THADA*] that were associated (including nominal associations) with all quantitative traits and/or overweight. Count and weighted risk scores (using trait specific β coefficients as weights) based on eight significant SNPs observed in the present study were fitted into the Fulker’s model for estimating within sib-pair effect (Table S6). The overall corrected p-value for risk score is 0.01 [35].

## Results and Discussion

Association of T2D related variants with various measures of obesity has been extensively evaluated among Europeans [19], [20], [36] but only few such studies exist in Indians [13], [15]. In the present study, we assessed the association of 25 such variants with overweight and five quantitative phenotypes related to obesity and found two of them to be associated with BMI, body fat and weight. In addition, we also found association of combined risk score with WHR and body fat after adjusting for BMI. All the tested SNPs were in HWE (Table S1) and the summary of characteristics (like age, sex, BMI, WHR, WC, glucose and insulin levels) of the study participants (normal, overweight and total IMS) are given in Table 1.

**Table 1:** Characteristics of Indian Migration Study Participants.

	Lean(N = 3304)		Overweight(3N = 1752)		Total IMS(N = 5056)	
	Mean	SD	Mean	SD	Mean	SD
**Age (in years)**	37.7	10.8	43.1	8.3	39.6	10.3
**Men (%)**	61.5	–	48.6	–	57.0	–
1 **BMI (kg/m** 2 **)**	21.0	2.6	29.3	3.0	23.5	4.5
2 **WHR**	0.85	0.07	0.88	0.08	0.86	0.08
**Waist Circumference (cm)**	76.0	9.1	91.0	9.3	81.8	11.6
**Body Fat**	22.8	7.3	29.8	7.3	26.8	8.2
**Weight (kg)**	54.3	8.9	71.6	9.8	60.3	12.4
**Fasting glucose (mmol/L)**	4.9	0.6	5.1	0.7	4.9	0.6
**Fasting insulin (mU/L)**	6.8	17.6	9.2	9.3	7.6	15.3

1 BMI: body mass index;

2 WHR: waist hip ratio;

3 IMS has 1752 overweight individuals out of 2666 individuals (1333 sib pairs concordant or discordant for overweight).

After FDR correction, we found associations of *CXCR4* rs932206 (A allele) and *HHEX* rs5015480 (C allele) with higher BMI (β = 0.13, standard error (se) = 0.04, p = 0.001), (β = 0.09, se = 0.03, p = 0.002), respectively, and with increased body fat (β = 0.10, se = 0.03, p = 0.0004), (β = 0.13, se = 0.04, p = 0.001), respectively (Table 2). The SNP at *HHEX* locus was also associated with body weight (β = 0.09, se = 0.03, p = 0.001) (Table 2). We have earlier showed an association of *CXCR4* with fasting glucose levels after adjusting for BMI in the IMS suggesting common loci explaining diabetes and obesity related traits [25]. Enough evidence exists that *HHEX* influences BMI in Europeans children [37]. Per allele, the *CXCR4* variant increases BMI by 0.58 kg/m^2^ and weight by 1.6 kg in the study population. Similarly, *HHEX* variant increases BMI by 0.40 kg/m^2^ and weight by 1.12 kg. This is substantially higher in comparison to a large scale investigation in the European population that found 0.17 kg/m^2^ increase in BMI (based on 32 loci related to BMI) for each unit increase in genetic susceptibility score [17]. We did not observe any changes in associations after adjusting for daily energy consumption and physical activity [Table S4] suggesting that their effects are not mediated through diet and physical activity.

**Table 2:** Within sib-pair association estimates for phenotypes related to obesity.

1SNP	Loci	Body Mass Index			Waist Circumference			Waist-hip Ratio			%Body fat			Weight		
		2β	3se	p	β	se	p	β	se	p	β	se	p	β	se	p
rs1799854	*ABCC8* (T)	−0.04	0.03	0.18	−0.04	0.03	0.19	−0.01	0.03	0.79	−0.02	0.02	0.43	−0.05	0.03	0.08
rs2641348	*ADAM30* (C)	−0.02	0.03	0.53	0.00	0.03	0.96	−0.02	0.03	0.58	0.00	0.03	0.90	−0.01	0.03	0.83
rs10490072	*BCL11A* (C)	−0.01	0.05	0.87	0.01	0.05	0.86	−0.01	0.04	0.78	−0.05	0.04	0.19	0.01	0.05	0.78
rs12779790	*CDC123* (G)	−0.05	0.04	0.86	0.03	0.04	0.51	0.05	0.04	0.18	0.00	0.03	0.99	0.00	0.04	0.91
rs7756992	*CDKAL1* (G)	−0.04	0.03	0.26	−0.04	0.03	0.19	0.01	0.03	0.71	−0.02	0.02	0.41	−0.04	0.03	0.20
rs10811661	*CDKN2A/B* (C)	−0.02	0.04	0.61	−0.02	0.04	0.61	0.01	0.04	0.85	−0.03	0.03	0.31	−0.03	0.04	0.37
rs932206	***CXCR4*** (A)	0.13	0.04	**0.001**	0.09	0.04	0.02	0.04	0.04	0.30	0.10	0.03	**0.0004**	0.13	0.04	**0.001**
rs1153188	*DCD* (A)	0.01	0.03	0.76	0.01	0.03	0.74	0.02	0.03	0.44	0.01	0.03	0.79	0.01	0.03	0.77
rs17044137	*FLJ39370* (A)	0.03	0.04	0.54	0.03	0.04	0.50	0.02	0.04	0.55	0.04	0.03	0.24	0.02	0.04	0.58
rs1055080	*FOXA2* (A)	0.11	0.05	0.01	0.03	0.05	0.57	0.00	0.04	0.92	−0.01	0.03	0.80	0.06	0.04	0.16
rs2268573	*GCK* (C)	0.02	0.03	0.55	0.01	0.03	0.79	0.01	0.03	0.66	0.00	0.02	0.87	0.02	0.03	0.51
rs5015480	***HHEX*** (C)	0.09	0.03	**0.002**	0.07	0.03	0.02	0.03	0.03	0.27	0.05	0.02	0.02	0.09	0.03	**0.001**
rs2237892	*KCNQ1* (T)	−0.02	0.12	0.90	−0.10	0.12	0.43	−0.11	0.12	0.34	−0.03	0.09	0.78	0.00	0.12	0.98
rs2876711	*KCTD12* (C)	0.01	0.03	0.77	−0.01	0.03	0.86	0.01	0.03	0.65	−0.04	0.02	0.07	−0.01	0.03	0.62
rs1256517	*LOC646279* (C)	−0.03	0.04	0.48	0.00	0.04	0.93	0.03	0.04	0.47	0.01	0.03	0.66	−0.03	0.04	0.43
rs10823406	*NGN3* (A)	0.03	0.03	0.31	0.06	0.03	0.09	0.08	0.03	0.01	0.05	0.02	0.06	0.03	0.03	0.38
rs10923931	*NOTCH2* (T)	0.01	0.03	0.76	0.03	0.03	0.43	0.00	0.03	0.93	0.02	0.03	0.56	0.02	0.03	0.59
rs1801282	*PPARG* (G)	0.05	0.04	0.22	0.04	0.04	0.36	0.02	0.04	0.60	−0.02	0.03	0.63	0.02	0.04	0.57
rs13266634	*SLC30A8* (T)	0.00	0.03	0.95	−0.01	0.03	0.65	0.01	0.03	0.83	0.00	0.03	0.86	−0.01	0.03	0.82
rs757210	*TCF2* (A)	−0.01	0.03	0.72	−0.03	0.03	0.30	−0.01	0.03	0.77	0.00	0.02	0.94	−0.02	0.03	0.46
rs7903146	*TCF7L2* (T)	−0.02	0.03	0.49	0.02	0.03	0.51	0.06	0.03	0.04	0.01	0.02	0.66	−0.02	0.03	0.55
rs7578597	*THADA* (C)	0.05	0.04	0.22	0.04	0.04	0.32	0.01	0.04	0.69	0.00	0.03	0.96	0.01	0.04	0.87
rs7961581	*TSPAN8, LGR5*(C)	0.00	0.03	0.96	−0.02	0.03	0.50	−0.02	0.03	0.57	0.02	0.02	0.38	−0.01	0.03	0.72
rs9472138	*VEGFA* (T)	−0.02	0.04	0.53	−0.05	0.04	0.18	−0.02	0.04	0.53	−0.04	0.03	0.12	−0.05	0.04	0.15
rs10010131	*WFS1* (A)	0.01	0.03	0.80	0.03	0.03	0.33	0.04	0.03	0.15	0.03	0.02	0.27	0.02	0.03	0.60

1 SNP: single nucleotide polymorphism;

2 β (Z score): within sib-pair coefficient of regression adjusted for age, sex and location;

3 se: standard error;

4 allele in parentheses indicate the risk allele.

We also observed nominal evidence of associations (p-values ranging from 0.01–0.04) of variants at *CXCR4* and *HHEX* with WC (p = 0.02 for both), predicting ∼0.8–1 cm increase in WC. *HHEX* variant was also associated with body fat (p = 0.02) with effective increase of 1% in body fat in absolute terms. Further, variation at *FOXA2* (rs1055060) was nominally associated (p = 0.01) with higher BMI and predicted an increase of 0.50 kg/m^2^ in BMI. This finding could be interesting for future exploration because loci that raise insulin levels are known to regulate BMI [21] and *FOXA2* plays an important role in improving insulin resistance [38]. In Indian population, *FOXA2* has been reported to be associated with higher fasting glucose, lower fasting insulin and risk for T2D [39]. Recently, association of *FOXA2* has also been detected in a genome-wide meta-analysis with fasting glucose among Europeans [21]. Further, *TCF7L2* rs7903146 (p = 0.04) and *NGN3* rs10823406 (p = 0.01) were weakly associated with increased WHR.

We found associations of variation at *CXCR4* and *HHEX* with overweight [OR = 1.7, 95% confidence interval (95%CI) = 1.2–2.3, p = 0.002 and OR = 1.5, 95%CI = 1.2–1.9, p = 0.002 respectively]. The high effect sizes, independent of dietary energy consumption and physical activity (Table S5), observed in the present study are not usually found in genetic epidemiological studies. In addition, weak associations between variation at *FLJ39307,* rs17044137, (p = 0.009); *LOC646279*, rs1256517, (p = 0.04) and *THADA*, rs7578597, (p = 0.03) with overweight were also observed. We have previously detected strong association between variation at *THADA* with T2D in the same population group even after adjusting for BMI [25], suggesting possible pleiotropic effects on overweight and T2D.

In further analyses, we incorporated all associated loci into an aggregate score to explore the combined effect of eight SNPs on obesity related phenotypes. The weighted risk score analysis showed evidence for association with WHR, which was consistent with the effects of single locus (0.81 SD increase per risk allele) (Table S6). The association with WHR suggests that the T2D related SNPs influence fat distribution more than overall adiposity. Further, it has been suggested that the genetic control for adiposity is different from mechanisms that influence BMI in Europeans [16]. In the present study, we adjusted the risk score for BMI and observed the association with WHR and body fat even after correcting for multiple testing. We also found nominal association of risk score with WC (p = 0.02). Our results support the evidence that fat distribution has different genetic basis than BMI and observations based on risk score analysis underline the possible role of all marginal associations in explaining obesity related quantitative traits which could individually be significant in a much larger study.

Previous sex specific analysis in Europeans has identified seven loci having marked differences in effect sizes for WHR suggesting gender specific differences in the regulation of body fat distribution [16]. We therefore explored sex-stratified analysis in the study population and found nominal evidence for non-differential association of *FLJ39370* in both males (OR = 2.02) and females (OR = 2.50) (Table S7). *CXCR4* variants also showed an effect of similar magnitude for overweight in both sexes (OR = 1.80), which was detectable only in the males but not in females, probably due to smaller sample size for females. In contrast, variants in *TCF2* (OR = 0.57, 95%CI = 0.35–0.95, p = 0.03) and *LOC646279* (OR = 0.29, 95%CI = 0.12–0.72, p = 0.007) were associated with protective effects against overweight in females with twice the effect size than in males (Table S7). These observations suggest sexual dimorphism of these effects, though results are not conclusive. *TCF2* was also associated with fasting insulin and homeostasis model assessment for insulin resistance (HOMA-IR) after adjustment for BMI indicating its potential for predicting traits related to obesity [25]. In formal tests for differences in the relationships between SNP and overweight by sex, there was no strong evidence of interaction; likely due to limited statistical power for interaction analysis.

The major strength of our study is the sib-pair design, which is resistant to population stratification and thus considerably lowers the possibility of false positive associations but at the cost of extra genotyping and power [40]. The present study confirms the value of evaluating T2D SNPs for obesity related traits in Indian population. In India, the availability of FFQ and physical activity data on large samples is rare. Therefore, opportunity of evaluating the mediation of effects of SNPs through daily energy consumption and levels of physical activity are additional strengths of the study.

The unavailability of genotype data on loci discovered in last two years [19], [20] is the major limitation of the study. Further, the lack of associations of the remaining SNPs might be because of limited power of our study for detecting small effect sizes. It could also be due to the fact that some of the analysed T2D SNPs were discovered from GWAS on lean subjects [36]. Another possible reason could be their small effect size or variability in linkage disequilibrium with the causal SNP in Indian population. We conducted an exploratory risk score analysis using a weighted risk score method because it is more powerful than the unweighted method. Due to the estimation of weights from the same data set in which the score was tested, our evaluation of weighted score may be biased. The unweighted score method has less of this bias but is not completely bias free. Finally, possible measurement error in FFQ like differences in reporting, overestimation of energy intake [41] could also be of concern while interpreting results adjusted for daily energy consumption.

In conclusion, we replicated the associations of T2D variants (*CXCR4* and *HHEX*) with BMI, body fat and weight, with larger effect size than in Europeans suggesting a common mechanism underlying T2D and measures of adiposity in Indian population. Further, the combined risk score predicting WHR and body fat, independent of BMI has strengthened the evidence that different genetic variants are possibly modulating fat distribution and BMI. This has also consolidated the case for evaluating the pooled effect of larger set of T2D related SNPs with measures of obesity. Our hypothesis of common biological underpinnings is further strengthened by the strong association of same T2D loci with overweight. Given the diversity in dietary pattern in India, the finding of associations not mediating through diet and identifying common loci for diabetes and obesity will have important implications especially for genotype and childhood BMI based early life interventions in south Asian region where obesity is a rapidly growing health problem.

## Supporting Information

Table S1 Hardy-Weinberg equilibrium in Indian Migration Study (IMS).
(DOCX)

Table S2 Allele frequencies, effect sizes and evidence of association of 25 loci considered for analysis.
(DOC)

Table S3 Power Calculations.
(DOC)

Table S4 Within sib-pair association estimates for phenotypes related to obesity adjusted for daily energy and physical activity.
(DOCX)

Table S5 Within sib-pair association estimates for overweight.
(DOC)

Table S6 Within sib pair effect of count and weighted risk score based on *8 significantly associated loci.
(DOC)

Table S7 Sex specific within sib pair effects for significantly associated SNPs with overweight.
(DOC)
