# Supplementary material for: Association Study of 25 Type 2 Diabetes Related Loci with Measures of Obesity in Indian Sib Pairs
Source: PLoS One. 2013 Jan 17;8(1):e53944. doi: 10.1371/journal.pone.0053944 (PMC3547960; doi:10.1371/journal.pone.0053944)
Supplement: Table S1 — Hardy-Weinberg equilibrium in Indian Migration Study (IMS). (DOCX) [file pone.0053944.s001.docx]

**Supplementary Information**

**Table-S1: Hardy-Weinberg equilibrium in Indian Migration Study (IMS)**

| **SNP** | **Loci** | **IMS (N-2528)**  ***^2^*p** |
| --- | --- | --- |
| rs1799854 | *ABCC8* | 0.201 |
| rs2641348 | *ADAM30* | 0.867 |
| rs10490072 | *BCL11A* | 0.102 |
| rs12779790 | *CDC123, CAMK1D* | 0.185 |
| rs7756992 | *CDKAL1* | 0.439 |
| rs10811661 | *CDKN2A/B* | 0.033 |
| rs932206 | *CXCR4* | 0.068 |
| rs1153188 | *DCD* | 0.375 |
| rs17044137 | *FLJ39370* | 0.005 |
| rs1055080 | *FOXA2* | 0.381 |
| rs2268573 | *GCK* | 0.124 |
| rs5015480 | *HHEX* | 0.124 |
| rs2237892 | *KCNQ1* | 0.024 |
| rs2876711 | *KCTD12* | 0.811 |
| rs1256517 | *LOC646279* | 0.526 |
| rs10823406 | *NGN3* | 0.865 |
| rs10923931 | *NOTCH2* | 0.069 |
| rs1801282 | *PPARG* | 0.131 |
| rs13266634 | *SLC30A8* | 0.720 |
| rs757210 | *TCF2* | 0.027 |
| rs7903146 | *TCF7L2* | 0.008 |
| rs7578597 | *THADA* | 0.065 |
| rs7961581 | *TSPAN8, LGR5* | 0.141 |
| rs9472138 | *VEGFA* | 0.451 |
| rs10010131 | *WFS1* | 0.030 |

^1^SNP: single nucleotide polymorphism; ^2^Corrected α<0.002
