# Supplementary material for: Association Study of 25 Type 2 Diabetes Related Loci with Measures of Obesity in Indian Sib Pairs
Source: PLoS One. 2013 Jan 17;8(1):e53944. doi: 10.1371/journal.pone.0053944 (PMC3547960; doi:10.1371/journal.pone.0053944)
Supplement: Table S2 — Allele frequencies, effect sizes and evidence of association of 25 loci considered for analysis. (DOC) [file pone.0053944.s002.doc]

**Table-S2: Allele frequencies, effect sizes and evidence of association of 25 loci considered for analysis**

| **^1^SNP** | **Loci** | **MAF-**  **(Risk allele)** | **MAF-**  **(Risk allele)** | **Effect Size**  **with T2D** | **Reference** | **Evidence for T2D and related traits in India** | **Association with**  **obesity traits** |
| --- | --- | --- | --- | --- | --- | --- | --- |
|  |  | **^2^GIH** | **^3^IMS** | **Europeans**  **^4^(95%CI)** |  |  |  |
| rs1799854 | *ABCC8* | 0.35 (A) | 0.32 (T) | 1.19 (**^5^**het) | Sladek et al. 2007 | ^6^Yes |  |
| rs2641348 | *ADAM30* | 0.23 (G) | 0.24 (C) | 1.10 (1.06-1.15) | Zeggini et al. 2008 | ^7^Yes | ^11^BMI |
| rs10490072 | *BCL11A* | 0.15 (C) | 0.09 (C) | 0.96 (0.91-1.02) | Saxena et al. 2007 |  |  |
| rs12779790 | *CDC123, CAMK1D* | n/a | 0.13 (G) | 1.14 (1.05‐1.24) | Voight et al. 2010 |  |  |
| rs7756992 | *CDKAL1* | 0.24 (G) | 0.26 (G) | 1.30 (1.19-1.42) | Cauchi et al. 2008 | ^8^Yes | ^12^Birth weight |
| rs10811661 | *CDKN2A/B* | 0.09 (C) | 0.15 (C) | 1.20 (1.14-1.25)` | Scott et al. 2007 | ^8^Yes |  |
| rs932206 | *CXCR4* | 0.16 (T) | 0.14 (A) | 1.18 (0.92–1.51) | Cauchi et al. 2008 | ^7^Yes |  |
| rs17044137 | *FLJ39370* | 0.12 (A) | 0.10 (A) | 1.13 (1.06–1.19) | Lewis et al. 2008 |  |  |
| rs1055080 | *FOXA2* | 0.10 (A) | 0.10 (A) | 0.68 (0.49-0.94) | Banasik et al. 2012 | ^9^Yes |  |
| rs5015480 | *HHEX* | 0.44 (C) | 0.45 (C) | 1.20 (1.12‐1.29) | Voight et al. 2010 | ^8^Yes | ^13^Pediatric BMI |
| rs2237892 | *KCNQ1* | 0.01 (T) | 0.01 (T) | 1.08 (1.06‐1.10) | Voight et al. 2010 | ^10^Yes |  |
| rs2876711 | *KCTD12* | 0.26 (C) | 0.30 (C) | 1.34 (1.21-1.48) | Scott et al. 2007 |  |  |
| rs1256517 | *LOC646279* | 0.10 (C) | 0.13 (C) | 1.11 (**^5^**het) | Sladek et a. 2007 |  |  |
| rs10823406 | *NGN3* | 0.27 (A) | 0.23 (A) | 1.19 (**^5^**het) | Sladek et al. 2007 |  |  |
| rs10923931 | *NOTCH2* | 0.22 (T) | 0.22 (T) | 1.17 (1.09‐1.26) | Voight et al. 2010 | ^7^Yes |  |
| rs1801282 | *PPARG* | 0.09 (G) | 0.13 (G) | 1.14 (1.08-1.20) | Scott et al. 2007 | ^8^Yes | ^14^Pediatric obesity |
| rs13266634 | *SLC30A8* | 0.22 (T) | 0.22 (T) | 1.12 (1.07-1.16) | Scott et al. 2007 | ^8^Yes | ^15^BMI |
| rs757210 | *TCF2* | n/a | 0.28 (A) | 1.12 (1.07-1.18) | Winckler et al. 2007 | ^7^Yes |  |
| rs7903146 | *TCF7L2* | 0.28 (T) | 0.29 (T) | 1.6 (**^5^**het) | Sladek et al. 2007 | ^8^Yes | ^15^BMI |
| rs7578597 | *THADA* | 0.17 (C) | 0.13 (C) | 1.23 (1.09‐1.41) | Voight et al. 2010 | ^7^Yes | ^11^BMI and ^11^WC |
| rs7961581 | *TSPAN8, LGR5* | 0.35 (C) | 0.33 (C) | 1.11 (1.04‐1.17) | Voight et al. 2010 |  |  |
| rs9472138 | *VEGFA* | 0.23 (T) | 0.16 (T) | 1.05 (0.96-1.14) | Scott et al. 2007 |  |  |
| rs10010131 | *WFS1* | 0.33 (A) | 0.26 (A) | 0.84 (0.77-0.92) | Sandhu et al. 2007 |  |  |

**^1^**SNP: Single nucleotide polymorphism; **^2^**GIH: Gujarati Indians in Houston, where, n/a=frequency is not available in HapMap data; **^3^**IMS: Indian Migration Study; **^4^**95%CI: 95% confidence interval; **^5^**het: heterozygous; **^6^**Chavali et al. (2011); **^7^**Gupta et al. (2012); **^8^**Chauhan et al. (2010); **^9^**Tabassum et al. (2008); **^10^**Been et al. (2011); **^11^**Staiger et al. (2008); **^12^**Zhao et al. (2009); **^13^**Zhao et al. (2010); **^14^**Dedoussis et al. (2009); **^15^**Dupuis et al. (2010)
