# Supplementary material for: Association Study of 25 Type 2 Diabetes Related Loci with Measures of Obesity in Indian Sib Pairs
Source: PLoS One. 2013 Jan 17;8(1):e53944. doi: 10.1371/journal.pone.0053944 (PMC3547960; doi:10.1371/journal.pone.0053944)
Supplement: Table S3 — Power Calculations. (DOC) [file pone.0053944.s003.doc]

**Table-S3: Power Calculations**

| **Software: Genetic Power Calculator**  [“[QTL association for sibships and singletons](http://pngu.mgh.harvard.edu/~purcell/gpc/qtlassoc.html)”] | | |
| --- | --- | --- |
| **Variation Explained**  **(N=2528)** | **Minor Allele Frequency** | |
|  | **10%** | **45%** |
|  | ***Power** | |
| **0.4%** | 53% | 53% |
| **0.5%** | 64% | 64% |
| **0.6%** | 72% | 72% |
| **0.7%** | 79% | 80% |
| **Sibling correlation 0.4* | | |
| **Software: Quanto 1.1**  [“case-sibling” option] | | |
| **Odds Ratio**  **(N=1333)** | **10%** | **45%** |
|  | ***Power** | |
| **1.2** | 30% | 65% |
| **1.3** | 56% | 92% |
| **1.4** | 78% | 99% |
