# Supplementary material for: Association Study of 25 Type 2 Diabetes Related Loci with Measures of Obesity in Indian Sib Pairs
Source: PLoS One. 2013 Jan 17;8(1):e53944. doi: 10.1371/journal.pone.0053944 (PMC3547960; doi:10.1371/journal.pone.0053944)
Supplement: Table S4 — Within sib-pair association estimates for phenotypes related to obesity adjusted for daily energy and physical activity. (DOCX) [file pone.0053944.s004.docx]

**Table-S4: Within sib-pair association estimates for phenotypes related to obesity adjusted for daily energy and physical activity**

| **^1^SNP** | **Loci** | **Body Mass Index** | | | **Waist Circumference** | | | **Waist-hip Ratio** | | | **%Body fat** | | | **Weight** | | |
| --- | --- | --- | --- | --- | --- | --- | --- | --- | --- | --- | --- | --- | --- | --- | --- | --- |
|  |  | **^2^β** | **^3^se** | **p** | **β** | **se** | **p** | **β** | **se** | **p** | **β** | **se** | **p** | **β** | **se** | **p** |
| rs1799854 | *ABCC8* | -0.04 | 0.03 | 0.18 | -0.04 | 0.03 | 0.16 | -0.01 | 0.03 | 0.72 | -0.02 | 0.02 | 0.38 | -0.05 | 0.03 | 0.08 |
| rs2641348 | *ADAM30* | -0.03 | 0.03 | 0.33 | -0.01 | 0.03 | 0.78 | -0.01 | 0.03 | 0.64 | -0.01 | 0.03 | 0.75 | -0.02 | 0.03 | 0.60 |
| rs10490072 | *BCL11A* | 0.00 | 0.05 | 0.92 | 0.02 | 0.05 | 0.70 | -0.01 | 0.04 | 0.86 | 0.05 | 0.04 | 0.14 | 0.01 | 0.05 | 0.78 |
| rs12779790 | *CDC123, CAMK1D* | 0.04 | 0.04 | 0.29 | 0.02 | 0.04 | 0.67 | 0.04 | 0.04 | 0.24 | -0.01 | 0.03 | 0.82 | -0.01 | 0.04 | 0.86 |
| rs7756992 | *CDKAL1* | -0.03 | 0.03 | 0.38 | -0.04 | 0.03 | 0.26 | 0.01 | 0.03 | 0.65 | -0.01 | 0.02 | 0.58 | -0.03 | 0.03 | 0.31 |
| rs10811661 | *CDKN2A/B* | -0.02 | 0.04 | 0.55 | -0.02 | 0.04 | 0.57 | 0.01 | 0.04 | 0.83 | -0.04 | 0.03 | 0.23 | -0.03 | 0.04 | 0.36 |
| rs932206 | ***CXCR4*** | 0.12 | 0.04 | **0.001** | 0.09 | 0.04 | 0.02 | 0.03 | 0.04 | 0.35 | 0.10 | 0.03 | **0.0004** | 0.12 | 0.04 | **0.001** |
| rs1153188 | *DCD* | 0.01 | 0.03 | 0.74 | 0.02 | 0.03 | 0.61 | 0.03 | 0.03 | 0.34 | 0.01 | 0.03 | 0.63 | 0.01 | 0.03 | 0.76 |
| rs17044137 | *FLJ39370* | 0.03 | 0.04 | 0.46 | 0.03 | 0.04 | 0.43 | 0.03 | 0.04 | 0.49 | 0.04 | 0.03 | 0.20 | 0.03 | 0.04 | 0.44 |
| rs1055080 | *FOXA2* | 0.11 | 0.05 | 0.02 | 0.02 | 0.05 | 0.59 | 0.00 | 0.04 | 0.93 | -0.01 | 0.03 | 0.80 | 0.06 | 0.04 | 0.20 |
| rs2268573 | *GCK* | 0.02 | 0.03 | 0.52 | 0.01 | 0.03 | 0.68 | 0.02 | 0.03 | 0.54 | 0.00 | 0.02 | 0.84 | 0.02 | 0.03 | 0.44 |
| rs5015480 | ***HHEX*** | 0.08 | 0.03 | **0.004** | 0.06 | 0.03 | 0.03 | 0.02 | 0.03 | 0.37 | 0.04 | 0.02 | 0.05 | 0.09 | 0.03 | **0.002** |
| rs2237892 | *KCNQ1* | -0.01 | 0.12 | 0.92 | -0.08 | 0.12 | 0.50 | -0.10 | 0.11 | 0.38 | -0.01 | 0.09 | 0.89 | 0.00 | 0.12 | 0.99 |
| rs2876711 | *KCTD12* | 0.01 | 0.03 | 0.65 | 0.00 | 0.03 | 0.96 | 0.01 | 0.03 | 0.64 | -0.04 | 0.02 | 0.07 | -0.01 | 0.03 | 0.78 |
| rs1256517 | *LOC646279* | -0.03 | 0.04 | 0.45 | 0.00 | 0.04 | 0.99 | 0.03 | 0.04 | 0.47 | 0.02 | 0.03 | 0.57 | -0.03 | 0.04 | 0.40 |
| rs10823406 | ***NGN3*** | 0.03 | 0.03 | 0.32 | 0.06 | 0.03 | 0.09 | 0.08 | 0.03 | **0.01** | 0.04 | 0.02 | 0.10 | 0.03 | 0.03 | 0.37 |
| rs10923931 | *NOTCH2* | 0.00 | 0.03 | 0.93 | 0.02 | 0.03 | 0.55 | 0.00 | 0.03 | 0.95 | 0.01 | 0.03 | 0.79 | 0.01 | 0.03 | 0.70 |
| rs1801282 | *PPARG* | 0.05 | 0.04 | 0.26 | 0.04 | 0.04 | 0.39 | 0.02 | 0.04 | 0.60 | -0.02 | 0.03 | 0.63 | 0.02 | 0.04 | 0.70 |
| rs13266634 | *SLC30A8* | 0.00 | 0.03 | 0.97 | -0.01 | 0.03 | 0.72 | 0.01 | 0.03 | 0.81 | 0.00 | 0.03 | 0.97 | 0.00 | 0.03 | 0.90 |
| rs757210 | *TCF2* | -0.01 | 0.03 | 0.65 | -0.03 | 0.03 | 0.29 | -0.01 | 0.03 | 0.78 | 0.00 | 0.02 | 0.93 | -0.02 | 0.03 | 0.43 |
| rs7903146 | ***TCF7L2*** | -0.01 | 0.03 | 0.65 | 0.02 | 0.03 | 0.41 | 0.06 | 0.03 | **0.04** | 0.01 | 0.02 | 0.53 | -0.01 | 0.03 | 0.67 |
| rs7578597 | *THADA* | 0.04 | 0.04 | 0.26 | 0.04 | 0.04 | 0.32 | 0.02 | 0.04 | 0.61 | 0.01 | 0.03 | 0.87 | 0.00 | 0.04 | 0.98 |
| rs7961581 | *TSPAN8,LGR5* | -0.01 | 0.03 | 0.84 | -0.03 | 0.03 | 0.38 | -0.02 | 0.03 | 0.46 | 0.02 | 0.02 | 0.48 | -0.02 | 0.03 | 0.58 |
| rs9472138 | *VEGFA* | -0.02 | 0.04 | 0.62 | -0.05 | 0.04 | 0.20 | -0.02 | 0.04 | 0.61 | -0.04 | 0.03 | 0.19 | -0.05 | 0.04 | 0.22 |
| rs10010131 | *WFS1* | 0.01 | 0.03 | 0.64 | 0.04 | 0.03 | 0.23 | 0.05 | 0.03 | 0.13 | 0.03 | 0.02 | 0.20 | 0.03 | 0.03 | 0.41 |

^1^SNP: single nucleotide polymorphism; ^2^β (Z score): within sib-pair coefficient of regression adjusted for age, sex, location, energy and daily average physical activity; ^3^se: standard error
