# Supplementary material for: Association Study of 25 Type 2 Diabetes Related Loci with Measures of Obesity in Indian Sib Pairs
Source: PLoS One. 2013 Jan 17;8(1):e53944. doi: 10.1371/journal.pone.0053944 (PMC3547960; doi:10.1371/journal.pone.0053944)
Supplement: Table S5 — Within sib-pair association estimates for overweight. (DOC) [file pone.0053944.s005.doc]

**Table-S5: Within sib-pair association estimates for overweight**

|  | **^1^SNP** | **Loci** | **Overweight** | | | **^4^Overweight (adjusted)** | | |
| --- | --- | --- | --- | --- | --- | --- | --- | --- |
|  |  |  | **^2^OR** | **^3^95%CI** | **p** | **OR** | **95%CI** | **p** |
| 1 | rs1799854 | *ABCC8* | 0.91 | 0.70-1.19 | 0.51 | 0.89 | 0.68-1.17 | 0.40 |
| 2 | rs2641348 | *ADAM30* | 0.81 | 0.61-1.07 | 0.14 | 0.75 | 0.56-0.99 | 0.05 |
| 3 | rs10490072 | *BCL11A* | 0.89 | 0.59-1.34 | 0.58 | 0.88 | 0.58-1.32 | 0.56 |
| 4 | rs12779790 | *CDC123, CAMK1D* | 1.20 | 0.88-1.78 | 0.20 | 1.27 | 0.89.1.82 | 0.20 |
| 5 | rs7756992 | *CDKAL1* | 0.84 | 0.64-1.11 | 0.23 | 0.84 | 0.64-1.11 | 0.23 |
| 6 | rs10811661 | *CDKN2A/B* | 0.99 | 0.71-1.38 | 0.97 | 0.97 | 0.69-1.36 | 0.85 |
| 7 | rs932206 | ***CXCR4*** | **1.60** | **1.19-2.28** | **0.003** | **1.69** | **1.22-2.35** | **0.00** |
| 8 | rs1153188 | *DCD* | 0.92 | 0.68-1.23 | 0.59 | 0.95 | 0.70-1.28 | 0.72 |
| 9 | rs17044137 | *FLJ39370* | 1.6 | 1.13-2.45 | 0.009 | 1.72 | 1.16-2.54 | 0.01 |
| 10 | rs1055080 | *FOXA2* | 1.46 | 0.99-2.14 | 0.05 | 1.46 | 0.99-2.16 | 0.06 |
| 11 | rs2268573 | *GCK* | 0.85 | 0.67-1.07 | 0.18 | 0.86 | 0.68-1.09 | 0.20 |
| 12 | rs5015480 | ***HHEX*** | **1.40** | **1.16-1.93** | **0.002** | **1.48** | **1.14-1.92** | **0.00** |
| 13 | rs2237892 | *KCNQ1* | 0.94 | 0.33-2.72 | 0.92 | 0.93 | 0.32-2.67 | 0.89 |
| 14 | rs2876711 | *KCTD12* | 0.89 | 0.69-1.15 | 0.41 | 0.94 | 0.73-1.21 | 0.63 |
| 15 | rs1256517 | *LOC646279* | 0.69 | 0.48-0.99 | 0.04 | 0.69 | 0.48-1.00 | 0.05 |
| 16 | rs10823406 | *NGN3* | 0.98 | 0.75-1.28 | 0.89 | 0.98 | 0.75-1.29 | 0.88 |
| 17 | rs10923931 | *NOTCH2* | 0.89 | 0.67-1.20 | 0.47 | 0.85 | 0.63-1.15 | 0.29 |
| 18 | rs1801282 | *PPARG* | 1.06 | 0.73-1.54 | 0.73 | 1.03 | 0.70-1.50 | 0.89 |
| 19 | rs13266634 | *SLC30A8* | 1.06 | 0.78-1.42 | 0.69 | 1.05 | 0.77-1.41 | 0.77 |
| 20 | rs757210 | *TCF2* | 0.89 | 0.69-1.16 | 0.42 | 0.88 | 0.68-1.14 | 0.34 |
| 21 | rs7903146 | *TCF7L2* | 0.85 | 0.65-1.10 | 0.22 | 0.85 | 0.66-1.11 | 0.24 |
| 22 | rs7578597 | *THADA* | 1.40 | 1.02-2.02 | 0.03 | 1.43 | 1.02-2.02 | 0.04 |
| 23 | rs7961581 | *TSPAN8, LGR5* | 1.06 | 0.83-1.36 | 0.58 | 1.07 | 0.84-1.37 | 0.58 |
| 24 | rs9472138 | *VEGFA* | 0.82 | 0.60-1.13 | 0.24 | 0.87 | 0.63-1.21 | 0.41 |
| 25 | rs10010131 | *WFS1* | 0.96 | 0.73-1.26 | 0.79 | 1.02 | 0.77-1.34 | 0.90 |

^1^SNP: single nucleotide polymorphism; ^2^OR= odds ratio; ^3^95%CI: 95% confidence interval; ^4^adjusted for age, sex, location, daily energy intake and daily average physical activity
