# Supplementary material for: Association Study of 25 Type 2 Diabetes Related Loci with Measures of Obesity in Indian Sib Pairs
Source: PLoS One. 2013 Jan 17;8(1):e53944. doi: 10.1371/journal.pone.0053944 (PMC3547960; doi:10.1371/journal.pone.0053944)
Supplement: Table S6 — Within sib pair effect of count and weighted risk score based on *8 significantly associated loci. (DOC) [file pone.0053944.s006.doc]

**Table-S6: Within sib pair effect of count and weighted risk score based on *8 significantly associated loci**

| **S. No.** | **Traits** | **Count risk score** | | | **Weighted risk score** | | | **Weighted risk score**  **(Adjusted for BMI)** | | |
| --- | --- | --- | --- | --- | --- | --- | --- | --- | --- | --- |
|  |  | **^1^β** | **^2^se** | **^3^p** | **β** | **se** | **^3^p** | **β** | **Se** | **^3^p** |
| 1 | Body mass index | 0.008 | 0.012 | 0.49 | 0.069 | 0.273 | 0.798 | - | - | - |
| 2 | Body fat | 0.011 | 0.009 | 0.20 | 0.372 | 0.209 | 0.07 | 0.355 | 0.151 | **0.019** |
| 3 | Weight | 0.001 | 0.012 | 0.95 | 0.056 | 0.268 | 0.832 | - | - | - |
| 4 | Waist circumference | 0.007 | 0.012 | 0.16 | 0.430 | 0.273 | 0.115 | 0.375 | 0.168 | 0.025 |
| 5 | Waist-Hip Ratio | 0.031 | 0.012 | **0.007** | 0.813 | 0.253 | **0.002** | 0.792 | 0.243 | **0.001** |

^1^β =regression coefficient; ^2^se=standard error; ^3^FDR corrected p value=0.01; *eight loci [*CXCR4, HHEX, FOXA2, NGN3, TCF7L2, FLJ39307, LOC646279* and *THADA*]
