# Supplementary material for: Association Study of 25 Type 2 Diabetes Related Loci with Measures of Obesity in Indian Sib Pairs
Source: PLoS One. 2013 Jan 17;8(1):e53944. doi: 10.1371/journal.pone.0053944 (PMC3547960; doi:10.1371/journal.pone.0053944)
Supplement: Table S7 — Sex specific within sib pair effects for significantly associated SNPs with overweight. (DOC) [file pone.0053944.s007.doc]

Table-S7: Sex specific within sib pair effects for significantly associated SNPs with overweight

|  | **^1^SNP** | **Loci** | **Males**  **[N=436 pairs]** | | | **Females**  **[N=331 pairs]** | | | **Interaction**  **p-value** |
| --- | --- | --- | --- | --- | --- | --- | --- | --- | --- |
|  |  |  | **^2^OR** | **^3^95%CI** | **^4^p** | **^2^OR** | **95%CI** | **^4^p** |  |
| 1 | rs932206 | *CXCR4* | **1.80** | 1.05-3.16 | **0.03** | 1.80 | 0.88-3.68 | 0.11 | 0.33 |
| 2 | rs17044137 | *FLJ39370* | **2.02** | 1.03-3.98 | **0.04** | **2.50** | 1.19-5.40 | **0.02** | 0.76 |
| 3 | rs5015480 | *HHEX* | 1.20 | 0.79-1.88 | 0.36 | 1.60 | 0.89-2.78 | 0.12 | 0.56 |
| 4 | rs1256517 | *LOC646279* | 0.73 | 0.40-1.33 | 0.31 | **0.29** | 0.12-0.72 | **0.007** | 0.48 |
| 5 | rs7578597 | *THADA* | 1.62 | 0.94-2.79 | 0.08 | 0.92 | 0.47-1.79 | 0.81 | 0.37 |
| 6 | rs757210 | *TCF2* | 0.91 | 0.57-1.44 | 0.69 | **.571** | 0.35-0.95 | **0.03** | 0.50 |

^1^SNP: single nucleotide polymorphism; ^2^OR= odds ratio adjusted for daily energy consumption; ^3^95%CI: 95% confidence interval; ^4^ FDR corrected p value is 0.008
